# Potential of Unmanned Aerial Vehicle Red–Green–Blue Images for Detecting Needle Pests: A Case Study with *Erannis jacobsoni* Djak (Lepidoptera, Geometridae)

**DOI:** 10.3390/insects15030172

**Published:** 2024-03-04

**Authors:** Liga Bai, Xiaojun Huang, Ganbat Dashzebeg, Mungunkhuyag Ariunaa, Shan Yin, Yuhai Bao, Gang Bao, Siqin Tong, Altanchimeg Dorjsuren, Enkhnasan Davaadorj

**Affiliations:** 1 College of Geographical Science, Inner Mongolia Normal University, Hohhot 010022, China; 20226016009@mails.imnu.edu.cn (L.B.); yinshan@imnu.edu.cn (S.Y.); baoyuhai@imnu.edu.cn (Y.B.); baogang@imnu.edu.cn (G.B.); tongsq223@imnu.edu.cn (S.T.); 2 Inner Mongolia Key Laboratory of Remote Sensing & Geography Information System, Inner Mongolia Normal University, Hohhot 010022, China; 3 Inner Mongolia Key Laboratory of Disaster and Ecological Security on the Mongolia Plateau, Inner Mongolia Normal University, Hohhot 010022, China; 4 Institute of Geography and Geoecology, Mongolian Academy of Sciences, Ulaanbaatar 15170, Mongolia; ganbat_d@mas.ac.mn (G.D.); mungunkhuyaga@mas.ac.mn (M.A.); 5 Institute of Biology, Mongolian Academy of Sciences, Ulaanbaatar 13330, Mongolia; altanchimeg_d@mas.ac.mn (A.D.); enkhnasand@mas.ac.mn (E.D.)

**Keywords:** *Erannis jacobsoni* Djak, UAV images, RGB vegetation indices, RGB texture features, machine learning, damage level recognition

## Abstract

**Simple Summary:**

An outbreak of the unique pest *Erannis jacobsoni* Djak in Mongolia would severely impact the forest ecosystem. Therefore, this study employed a combination mode of UAV-RGB vegetation indices and texture features, utilizing the sequential projection algorithm to extract sensitive features and machine learning algorithms to construct a damage level recognition model, achieving low-cost, rapid, and effective pest detection. The results indicate that the combined mode of the RGB vegetation indices and texture features yielded good pest detection results, with an overall accuracy of 89%. This could provide an important experimental foundation for subsequent large-scale forest pest monitoring with a high spatiotemporal resolution.

**Abstract:**

*Erannis jacobsoni* Djak (Lepidoptera, Geometridae) is a leaf-feeding pest unique to Mongolia. Outbreaks of this pest can cause larch needles to shed slowly from the top until they die, leading to a serious imbalance in the forest ecosystem. In this work, to address the need for the low-cost, fast, and effective identification of this pest, we used field survey indicators and UAV images of larch forests in Binder, Khentii, Mongolia, a typical site of *Erannis jacobsoni* Djak pest outbreaks, as the base data, calculated relevant multispectral and red–green–blue (RGB) features, used a successive projections algorithm (SPA) to extract features that are sensitive to the level of pest damage, and constructed a recognition model of *Erannis jacobsoni* Djak pest damage by combining patterns in the RGB vegetation indices and texture features (RGB_VI&TF_) with the help of random forest (RF) and convolutional neural network (CNN) algorithms. The results were compared and evaluated with multispectral vegetation indices (MS_VI_) to explore the potential of UAV RGB images in identifying needle pests. The results show that the sensitive features extracted based on SPA can adequately capture the changes in the forest appearance parameters such as the leaf loss rate and the colour of the larch canopy under pest damage conditions and can be used as effective input variables for the model. The RGB_VI&TF_-RF_440_ and RGB_VI&TF_-CNN_740_ models have the best performance, with their overall accuracy reaching more than 85%, which is a significant improvement compared with that of the RGB_VI_ model, and their accuracy is similar to that of the MS_VI_ model. This low-cost and high-efficiency method can excel in the identification of *Erannis jacobsoni* Djak-infested regions in small areas and can provide an important experimental theoretical basis for subsequent large-scale forest pest monitoring with a high spatiotemporal resolution.

## 1. Introduction

*Erannis jacobsoni* Djak (Lepidoptera, Geometridae) is a unique leaf-feeding pest in Mongolia that feeds on larch needles, and it causes the most damage during its larval stage (June to July) [1]. During this period, larvae violently feed on needles, causing larch to slowly shed from the top and the growth condition of the trees to gradually suffer until death, which leads to a serious imbalance in the forest ecosystem [2]. According to a survey, since 1920, *Erannis jacobsoni* Djak has shown a trend of spreading from the northwest to the southeast of Mongolia, and the typical outbreak area of the Khentii province is only over a hundred kilometres away from China’s Greater Khingan Mountains’ forest area [3]. Since there is no natural barrier between the two countries for interception, pest invasion is very likely. The pest has a strong adaptability to the environment; once it invades new areas, it will easily form a dominant population, which will cause immeasurable environmental damage to forest areas and economic losses. It is evident that the timely monitoring and control of this pest are extremely important to protect forest ecosystems. At present, pest prevention measures in Mongolia are based on the manual dispersal of chemical pesticides or biological pesticides [4], which are implemented mainly by experience, do not distinguish among pest distribution areas, and lack a precise guidance basis [5], resulting in the insufficient application of pesticides to severely affected areas and their excessive application to mildly affected areas, leading to environmental pollution [6]. Therefore, methods that identify the level of pest damage for *Erannis jacobsoni* Djak can not only improve the efficiency of pesticide implementation and reduce the pollution of the environment by pesticides but also maintain the balanced development of plant ecosystems, which has theoretical significance and practical value in maintaining ecological security.

Research on pest damage monitoring has been the focus of scholars both domestically and internationally [7,8]. In traditional pest research, pest monitoring and investigation are mainly carried out by professionals on-site, resulting in relatively accurate and reliable data. However, this method is time-consuming, labour-intensive, expensive, and environmentally destructive and cannot meet the demands of large-scale applications. The development of remote sensing technology has made it possible to monitor pest damage at a regional scale [9,10]. Over the past few decades, satellite remote sensing technology has developed significantly, achieved a high monitoring accuracy, and been widely applied by scholars [11,12,13]. However, the potential applications of satellite remote sensing in many pest research areas have been limited by low temporal and spatial resolutions, high costs, and weather conditions [14]. In addition, many monitoring models can only provide high-precision experimental results at a large scale, such as at the national, provincial, or municipal level, and cannot describe the changes in pest infestation in detail within relatively small areas [15].

Recently, the utilisation rate of unmanned aerial vehicle (UAV) platforms has increased. Their advantages, such as ease of operation, high spatial resolution, and high observation frequency, have shown to be of practical value in natural disaster research [16,17]. The calculation of vegetation indices based on UAV imagery spectral reflectance has been proven to be an effective method for monitoring the level of plant damage. For example, Abdollahnejad et al. used dual-temporal UAV data to calculate vegetation indices and assessed the health of mixed broad-leaved and needle-leaved forests using machine learning algorithms [18]. Ma et al. combined spectral information based on UAV multispectral data and applied deep learning methods to invert the damage information of *Tomicus yunnanensis* [19]. Guerra-Hernández et al. discriminated the level of damage of black alder under *Phytophthora* infestation using UAV multispectral vegetation indices, achieving a maximum accuracy rate of 75% [20]. The above research confirms that, compared with traditional methods, appropriate spectral vegetation indices can be used to more effectively monitor pests and diseases, but this requires more advanced and expensive multispectral sensors as a basis. As an alternative, some scholars have investigated vegetation parameters and pests and diseases using vegetation indices obtained from the red–green–blue (RBG) images of commercial unmanned aerial vehicle RGB cameras [21]. For example, del-Campo-Sanchez et al. used UAV RGB imagery to detect the damage level of *Jacobiasca lybica* pests in vineyards [22]. De Castro et al. differentiated healthy and wilt-diseased avocado trees using vegetation indices calculated from UAV RGB images and obtained good results [23]. Although the cost of acquiring data from UAV RGB vegetation indices (RGB_VI_) is relatively low, the monitoring effect is comparatively inferior to that of multispectral data [24,25], and its accuracy cannot fully meet the requirements. Therefore, if other appropriate features are added on the basis of RGB_VI_, it may be possible to achieve an improvement in detection accuracy [26]. The texture structure information of the tree canopy, which is widely recognised as a characteristic quantity in addition to spectral information, can reflect features that cannot be reflected by the spectrum. It is also one of the factors that affect the robustness of vegetation indices [27,28] and can be used as an ideal feature combined with RGB_VI_. It can reflect the subtle changes in trees with insect pests, avoid the influence of factors such as “same spectrum, different object” and “same object, different spectrum” in the presence of land features, and stretch the distance of an image [29]. It has great potential in plant pest identification and increases the feasibility of some studies. Currently, there are more cases of using RGB texture features (RGB_TF_) alone to monitor vegetation [30,31] than there are reports on using RGB_TF_ with other data as monitoring variables. The complementary fusion of RGB_VI_ and RGB_TF_ features can unlock the unlimited potential of RGB images for pest observation, opening up new economic, high-frequency, and high-precision ways to monitor pests. This is of great significance for the rapid diagnosis and prevention of *Erannis jacobsoni* Djak pest infestation.

In terms of vegetation health detection methods, scholars have used algorithms that focus on traditional machine learning and deep learning. For example, Syifa et al. used two machine learning algorithms, that is, support vector machines and artificial neural networks, to distinguish between healthy and affected trees in the case of pine wilt disease, achieving an accuracy of 94.13% [32]. Duarte et al. utilised the random forest algorithm to detect the damage status of eucalyptus trees under the threat of eucalyptus long-horned borers, achieving a classification accuracy of 98.5% [33]. Liu et al. identified 31 categories of forestry pests using the YOLO-4 algorithm with convolutional neural networks and obtained excellent results [34]. Among them, the random forest (RF) and convolutional neural network (CNN) classifiers are frequently and widely used for quantitative tree and vegetation pest detection due to their excellent computing speed and ability to handle complex data, respectively [35,36,37].

Based on the above discussion, the purpose of this paper is to identify the damage level of *Erannis jacobsoni* Djak based on UAV remote sensing images of typical areas of *Erannis jacobsoni* Djak infestation, combined with RGB_VI_ and RGB_TF_ information, using RF and CNN methods to answer three basic questions: (i) whether the successive projections algorithm (SPA) can filter out the features that are sensitive to the level of pest damage from many variables, (ii) whether the combined pattern of RGB_VI_ and RGB_TF_ (RGB_VI&TF_) can improve the accuracy of the pest detection model, and (iii) how to choose a suitable model to build the algorithm when the sample size is unstable.

## 2. Materials and Methods

### 2.1. Study Area

**Figure 1:**
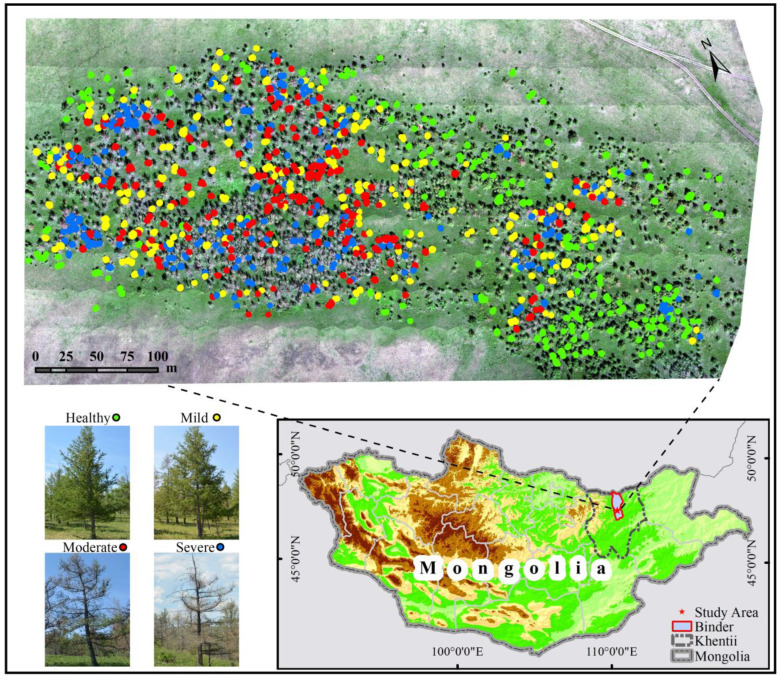
Location of the study area and sample trees.

The study area is located within the typical outbreak area of *Erannis jacobsoni* Djak: a region of Binder, Khentii, Mongolia, with a length of 600 m, a width of 300 m and an average altitude of 1100 m. The study area was mainly dominated by *Larix sibirica*, with approximately three thousand *Larix sibirica* with different levels of damage distributed in the area, and the tree species was relatively homogeneous, which provided natural conditions for the invasion of *Erannis jacobsoni* Djak. The area had been frequently infested with *Erannis jacobsoni* Djak between 2010 and 2020, and signs of the pest were found by the local forestry survey team in late May and early June 2021. Therefore, the researchers used an UAV to collect RGB visible and multispectral image data from the test area in late June 2021. Meanwhile, 840 larch sample trees were randomly selected from the test area for the study on pest damage level identification (Figure 1).

### 2.2. Materials Acquisition and Processing

#### 2.2.1. Field Data

In the study area, 840 sample trees of larch with different levels of damage were selected, and a survey of the geospatial coordinates and leaf loss rate of each tree was completed. The sample trees were divided into three layers—upper, middle, and lower—and three typical branches of each layer were selected to count healthy and damaged needles and calculate the leaf loss rate using Equation (1). Then, the average value was taken as the leaf loss rate of the current sample tree.

(1) $$DR=\frac{L_{d}}{L_{h}+L_{d}}\times 100\%$$

where DR denotes the rate of leaf loss and takes values between 0 and 100%, and L_h_ and L_d_ denote the number of healthy and damaged needles, respectively. On this basis, through the experience of visual discrimination in field and the classification criteria in previous studies, the results were classified into pest damage levels based on Table 1 [38], where the final classification results of 210 larch trees into healthy, mild, moderate, and severe levels are shown.

#### 2.2.2. UAV Image Data

**Figure 2:**
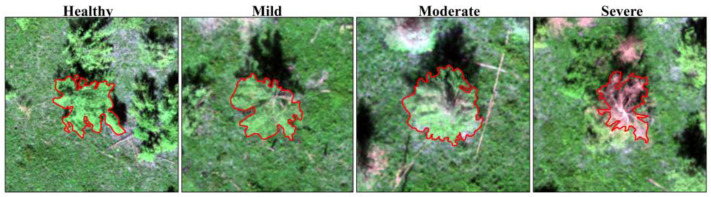
Tree canopy vectorisation (red border) and damage level assignment.

A test was conducted with a DJI Phantom 4 multispectral quadcopter drone equipped with an all-in-one imaging system with RGB visible sensors (red, green, and blue channels) and five multispectral sensors (blue, green, red, red-edge, and near-infrared bands). Each camera had a 200-pixel resolution, and the resolution reached the centimetre level. Data acquisition was conducted under clear, cloudless, and windless conditions from 10:00 to 14:00 British Summer Time (BST), with the flight altitude being set at 100 m. The camera was calibrated with a whiteboard before the flight, and the camera probe went down vertically during the flight to acquire the observation images. After the flight, the images were preprocessed by “DJI Terra” to obtain two types of images—RGB and multispectral images. On this basis, the sample larch was visually segmented with ArcMap10 to obtain the canopy vector, and the damage level was assigned according to the measured leaf loss rate data (Figure 2).

#### 2.2.3. Feature Extraction

Vegetation indices are combinations of two or more reflectance wavelengths that enhance differences in reflectance characteristics between stands of various levels of damage and are less influenced by light and background [39]. Referring to a previous study, we used 60 multispectral vegetation indices (MS_VI_) and 22 RGB vegetation indices (RGB_VI_), which are widely used in plant pest and related studies, for the detection of *Erannis jacobsoni* Djak pests [5,15,18,25,40,41,42]. First, the MS_VI_ and RGB_VI_ were calculated from the corresponding images using “Envi”. Second, based on the sample tree canopy vector, the average value of each feature was extracted tree-by-tree as the index value of the sample tree at hand.

In addition, to extract RGB_TF_, principal component analysis (PCA) was performed based on the red, green, and blue channels of the RGB images. PCA is a statistical method to transform a set of potentially correlated variables into a set of linearly uncorrelated variables by orthogonal transformation, which can reduce the dimensionality of the feature space to achieve the elimination of redundant information, help speed up the calculation, and improve the accuracy of a model [26]. Among various texture extraction algorithms, the grey-level cogeneration matrix (GLCM) is widely used for texture analysis [43]. The results of the PCA were used to calculate eight texture feature values by GLCM, including the mean (mean), variance (var), homogeneity (hom), contrast (con), dissimilarity (dis), entropy (ent), second moment (sm), and correlation (corr) values [44,45], and the mean value of each feature was calculated as the RGB_TF_ value of each sample tree by the sample trees canopy vector on a tree-by-tree basis. The final calculation results of MS_VI_, RGB_VI_, and RGB_TF_ were normalised to reduce errors.

### 2.3. Methods

#### 2.3.1. Feature Sensitivity Analysis and Extraction

(1) Sensitivity analysis of variance

An analysis of variance (ANOVA) is used to determine whether subtyped independent variables have a significant effect on numerical dependent variables by calculating the variance statistic F value and testing whether the means of each aggregate are equal. The basic idea is to determine the magnitude of the influence of controllable factors on the study results by analysing the magnitude of the contribution of variance from different sources to the total variance. With the help of ANOVA, the variances of MS_VI_, RGB_VI_, and RGB_TF_ on the pest damage level were calculated to reveal the sensitivity of these features to the pest damage level. The larger the F value of a feature, the more significant the sensitivity of the feature to the level of pest damage.

(2) Sensitive feature extraction

With the help of SPA, MS_VI_, RGB_VI_, and RGB_VI&TF_ were downscaled to eliminate overlapping and redundant information and retain meaningful features, which were set as the sensitive feature sets of MS_VI_, RGB_VI_, and RGB_VI&TF_ as the input variables of the recognition model. SPA is a forward variable selection algorithm that minimises the covariance of modelling variables. It has the advantage that extracting a few columns of data in the initial data set can summarise the information of the vast majority of feature variables, achieve the elimination of redundant information, minimise information overlap, perform well when dealing with large-scale data, filter modelling features, and improve model accuracy. Details of the SPA algorithm can be found in [2].

#### 2.3.2. Multispectral and RGB Features for Needle Pest Recognition

(1) Pest recognition model with sensitive features

By combining the damage level of the pests to the sampled larch and the corresponding sensitive feature sets, the damage level recognition model of *Erannis jacobsoni* Djak was established in MATLAB2022 with the help of RF and CNN algorithms, which were used to explore the application potential of sensitive features. RF is a data mining model that is commonly used for classification prediction. Its main principle is to generate a new set of training samples by repeatedly randomly sampling k samples from the original training sample set of a size N through the bootstrap resampling technique and then generate k classification trees to form a random forest based on the self-help sample set. The classification results of the new data are determined by the number of votes formed by the classification trees to obtain a more accurate and stable prediction result [46]. CNN is widely used in deep learning and is a deep neural network with a convolutional structure. The model usually consists of five parts: input, convolution, pooling, dense connection, and output. It can map high-dimensional nonlinear data to a low-dimensional space, realise data dimensionality reduction, effectively reduce the number of parameters in the network, and alleviate the overfitting problem of the model [38].

(2) Analysis of the pest recognition potential of sensitive features

A total of 75% of the trees from all the samples were randomly selected as the training data set (including a training set and a validation set) for modelling and optimal model selection, and the remaining 25% were used as the test data set to validate the model and analyse its pest recognition potential. To objectively evaluate the model’s performance, the overall accuracy (OA), Kappa coefficient, and confusion matrix were calculated based on true positives (TP), false positives (FP), true negatives (TN), and false negatives (FN), the main metrics for model accuracy validation [21,47,48]. OA is the probability that the classification result for each random sample is consistent with the type of data tested, ranging from 0 to 1. A larger value indicates a higher accuracy of the model’s implementation. The Kappa coefficient is a metric used for consistency testing and represents the proportion of error reduction generated by classification versus completely random classification; it ranges from −1 to 1. Larger values indicate a better stability of the model’s implementation and vice versa. The confusion matrix is calculated by comparing the victimisation level of each measured sample with the corresponding victimisation level after prediction classification, which can characterise the classification accuracy of the model for each victimisation level in this paper; the user accuracy (UA) and producer accuracy (PA) in the confusion matrix are used to evaluate the discrimination results of each damage level. The specific formulas for the accuracy evaluation metrics above are as follows:

(2) $$OA=\frac{TP+TN}{TP+TN+FP+FN}$$

(3) $$Kappa=\frac{OA-\frac{\sum_{i=1}^{k}N_{P}\times N_{t}}{S^{2}}}{1-\frac{\sum_{i=1}^{k}N_{P}\times N_{t}}{S^{2}}}$$

where k is the number of classes, N_p_ is the number of predictions, N_t_ is the number of actual measurements, and S is the sample size.

## 3. Results

### 3.1. Sensitivity Analysis of RGB Features

**Figure 3:**
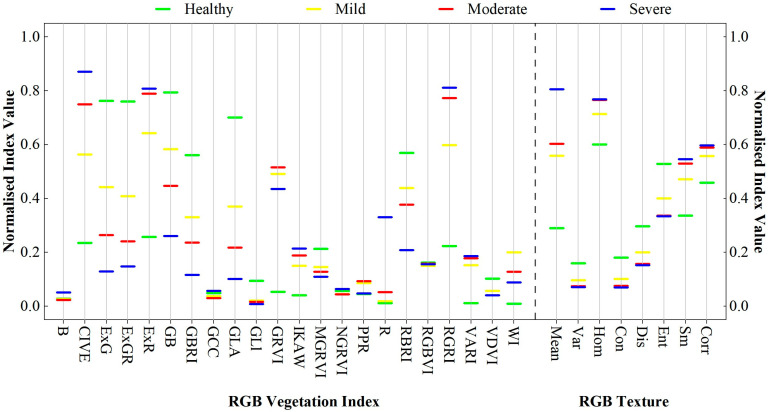
The distribution of tree vegetation indices at different damage levels.

To investigate the response of RGB characteristics to different damage levels, four damage levels of larch and the corresponding RGB features were plotted (Figure 3). As shown in the figure, most of the features showed significant hierarchical changes in the level of damage, from healthy to severe. Specifically, the indices CIVE, ExR, R, RGRI, and VARI showed a gradual increase; the indices B, GCC, GRVI, NGRVI, PPR, and WI showed irregularities; and the remaining indices showed a decreasing trend. For RGB_TF_, the mean, hom, sm, and corr showed upwards trends, and the var, con, dis, and ent showed downwards trends. This is because, when the larch is healthy, its leaf loss rate is minimal, the biochemical fraction of needles is sufficient, the canopy appears green, and all the information reflected in the RGB images is normal vegetation information. When the pests begin to invade larch, abnormal changes in the content of biochemical components of the needles, an increase in the rate of leaf loss in the stand, and a change in the canopy colour from green to yellow, red, and grey, which, in turn, affect the information captured by the RGB channel, are obvious responses to changes in the level of pest damage, implying that it is feasible to use RGB features to identify the degree of pest damage.

**Figure 4:**
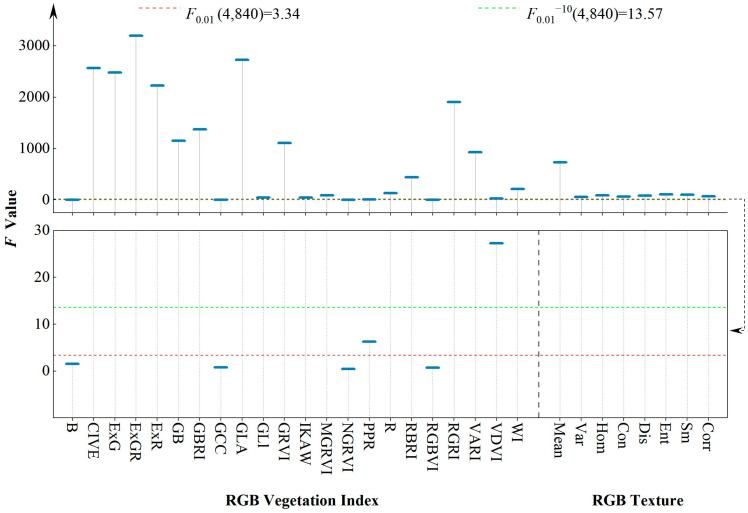
Variance of RGB features.

To reveal the sensitivity of the selected features to different damage levels of the pest, the damage levels were subjected to ANOVA with the corresponding RGB features, and the variance distribution was plotted (Figure 4). As it can be seen from the figure, the condition under which the variance of different damage levels and each feature satisfies *p* < 0.01 is F > F_0.01_ (4, 840), indicating that features with F > 3.34 are sensitive to different damage levels of the pest. When F > F_0.01_^−10^ (4, 840), *p* < 0.01^−10^, indicating that the sensitivity between the features with an F value larger than 13.57 and the level of pest damage is extremely significant [49]. The results showed that all the features except for B, GCC, NGRVI, and RGBVI were highly sensitive with respect to the degree of damage. All the features except for PPR had an F value of 13.57, with significant sensitivity to different damage levels of the pest. For RGB_VI_, the index ExGR had the highest F value of 3194.7, while, in RGB_TF_, the mean reached the highest value (F = 731.8). It is evident that most of the features analysed were significantly sensitive to different damage levels of the pests and had a good ability to identify the damage level.

### 3.2. Sensitive Feature Extraction

The recognition model was constructed by extracting sensitive input variables through the SPA algorithm, and then, based on the optimal model, the sensitive features were analysed, and their feature contributions were obtained.

The results show that, for the optimal RF model (Table 2), seven features of all MS_VI_s were selected as sensitive features. Among them, the NDVIreg, SI1reg, SI1reg*, and TCARI indices had a red-edge band in their constituent spectral bands because the red-edge band was in the middle of the red-valley and near-infrared (NIR) bands, and its changes were influenced by the simultaneous reflectance of the red-valley and near-infrared bands that are correlated with the degree of pest damage, which is the fastest changing band in the red-edge region [50]. The presence of NIR bands in the constituent spectral bands of the 2NLI, GDVI, and GMNLI indices was due to the fact that the NIR bands were controlled by parameters such as plant moisture and internal structure that had a significant response to the level of stand damage. The RGB_VI_ and RGB_VI&TF_ sensitive feature sets included nine and thirteen features, respectively, of which eight features were selected in both feature sets. When RGB_TF_ was added to RGB_VI_, slightly more features were selected by SPA, especially the four selected in RGB_TF_, indicating that the addition of texture features added necessary information to the identification of pest damage levels based on the RGB features.

For the CNN optimal model (Table 3), the sensitive feature set of MS_VI_ included ten indices, of which seven indices had red-edge bands in the constituent spectral bands, and three indices had NIR bands in the constituent spectral bands. The sensitive feature sets of RGB_VI_ and RGB_VI&TF_ contained four and six features, respectively, and three of them were selected by both feature sets. In the results, the index GLA could handle the brightness of the vegetation cover images, which could attenuate the interference of shadows between the trees and, thus, reflect the change in the vegetation canopy [51]. The indices GBRI, RBRI, and RGRI could describe and analyse the angular sensitivity of the vegetation indices, which could effectively deal with the complex vegetation canopy structure and were more sensitive to the rate of needle leaf loss during the damage process [25,52]. The index GB could enhance the difference in spectral response between the blue and green channels and could characterise the information of conifer chlorophyll content in the forest trees [53]. The dis feature was able to reflect the degree of inhomogeneity between image elements and had excellent results in edge detection, helping researchers identify complex canopy appearance shapes [54]. The texture feature mean had a good ability to classify pest damage areas [29].

**Figure 5:**
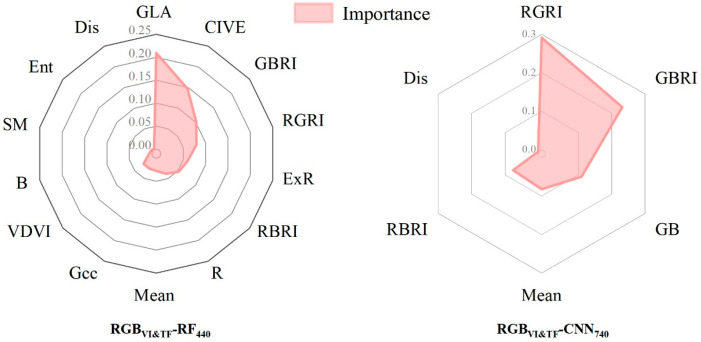
The importance of sensitive RGB features for optimal RGB_VI&TF_ models.

In addition, in order to understand the influence of the input features on the model, the contribution of sensitive features was calculated to reveal the importance of each variable in pest recognition. The main purpose of this study was to demonstrate the pest recognition potential of the RGB_VI&TF_ model, so the RF and CNN optimal models based on RGB_VI&TF_ were used to calculate the contribution of each sensitive feature with the help of the Gini coefficient, as shown in Figure 5. The RF optimal model had the largest contribution of GLA to the model at 0.21, indicating that this feature provided more decisive information for the model. It was followed by CIVE, GBRI, RGRI, and ExR. The contribution of RGB_TF_ was relatively low compared to RGB_VI_, with mean, sm, ent, and dis values of 0.03, 0.004, 0.003, and 0.0008, respectively. The contributions of RGRI and GBRI in the CNN optimal model were high, at 0.29 and 0.23, respectively, while the contribution of other vegetation indices was 0.11 for GB and 0.08 for RBRI. The contributions of the texture features mean and dis were 0.08 and 0.0009, respectively. The contribution of the mean in the texture features was significant and it was robust to outliers and could reflect global pest occurrence; thus, it is a universal feature and has the potential to be applied to pest recognition research.

### 3.3. Analysis of the Pest Damage Level Recognition Potential of RGB Features

#### 3.3.1. Overall Accuracy Evaluation of Pest Damage Recognition

Based on the sensitive features of RGB_VI&TF_ extracted by the SPA algorithm, we constructed the recognition model of *Erannis jacobsoni* Djak pest damage degree with the help of CNN and RF algorithms under the condition of different numbers of sample trees, verified its accuracy (Table 4 and Table 5), and compared and evaluated the recognition results with the sensitive features of MS_VI_ and RGB_VI_.

As seen from the table, among the RF models, MS_VI_-RF, RGB_VI_-RF, and RGB_VI&TF_-RF showed a gradual increase in accuracy as the number of sample trees increased gradually from 140 until the OA and Kappa of all three models (MS_VI_-RF_440_, RGB_VI_-RF_440_, and RGB_VI&TF_-RF_440_) peaked when the number of samples reached 440 and became the optimal model, at which point the accuracy began to decrease gradually. This indicated that the best model performance for studies applying RF to recognise the damage level of *Erannis jacobsoni* Djak was achieved with a sample size of approximately 440. Among these optimal models, MS_VI_-RF_440_ achieved the highest OA and Kappa values of 0.9091 and 0.8843, which were improvements compared to the 0.0636 and 0.0751values, respectively, obtained by RGB_VI_-RF_440_. This was because spectral reflectance was more responsive to subtle changes in covariates such as vegetation chlorophyll and leaf loss rate than R, G, and B, which were sensitive only to the canopy colour of affected larch. To improve the recognition effect of RGB_VI_, it was combined with RGB_TF_, and this method achieved a significant improvement in model accuracy. Specifically, compared to RGB_VI_-RF_440_, the OA and Kappa of RGB_VI&TF_-RF_440_ improved by 0.0181 and 0.0203, respectively, and the difference between the OA and Kappa of MS_VI_-RF_440_ was reduced by 0.0455 and 0.0548, respectively.

For the CNN model, the accuracy also improved when increasing the number of sample trees from 140 to 840. The OA and Kappa coefficients of the three models reached their highest values when the number of samples was 740; these were set as the optimal models (MS_VI_-CNN_740_, RGB_VI_-CNN_740_, and RGB_VI&TF_-CNN_740_). This suggests that, when recognizing *Erannis jacobsoni* Djak damage levels with CNN, the training sample can be increased as much as possible to improve the model accuracy, and the sample size is most suitable at approximately 740. Among the three models, MSVI-CNN740 achieved the highest accuracy with OA and Kappa coefficients of 0.9135 and 0.8892, respectively, which were 0.0486 and 0.0586 higher than those of RGB_VI_-CNN_740_. By combining RGB_VI_ and RGB_TF_ features, optimisation of the model was achieved. Specifically, the OA and Kappa coefficients of RGB_VI&TF_-CNN_740_ improved by 0.0216 and 0.0259, respectively, compared to RGB_VI_-CNN_740_, and the difference between them and MS_VI_-CNN_740_ was reduced to 0.027 and 0.0327. These results are consistent with most scholars’ findings [55,56,57].

#### 3.3.2. Accuracy Evaluation of Different Damage Levels Recognition

**Figure 6:**
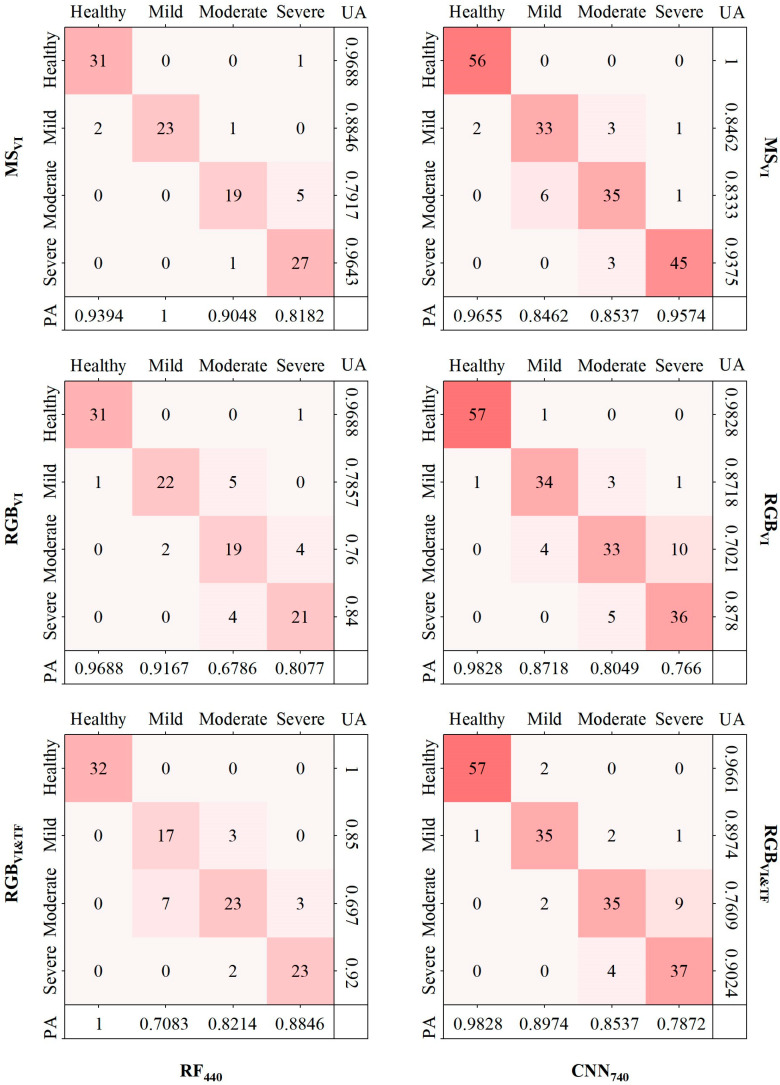
Confusion matrices of different classification models.

To explore the discriminative effect of the model on each class in more detail, the confusion matrix of the optimal model was drawn by combining the results of the actual measurements and predictions (Figure 6).

As seen from the figure, all the models showed excellent results in the discrimination of healthy stands, followed by the discrimination of the level of severe damage, with UA being more prominent. In the RF model, the discrimination of healthy and severely damaged stands was improved more by considering RGB_VI&TF_ compared to RGB_VI_, especially in the discrimination of healthy stands, where both UA and PA reached a value of 1, indicating that there were no commission and omission. In the CNN model, compared to RGB_VI_, RGB_VI&TF_ improved the discrimination of mild, moderate, and severe damage stands, with UA and PA improving by 0.0256, 0.0588, and 0.0244 and 0.0257, 0.0488, and 0.0212, respectively, while its UA decreased with respect to the discrimination of healthy stands due to two trees being misclassified as healthy larch. The above results show that the combination variables of RGB_VI_ and RGB_TF_ had substantial correlations with the level of tree damage and had great application effect and value for *Erannis jacobsoni* Djak pest recognition efforts.

## 4. Discussion

### 4.1. Efficiency of SPA-Based Selection of Sensitive Features

In some previous plant pest studies, scholars modelled all selected features to monitor the severity or physicochemical parameter content [58], but not all the features have a positive effect on the study process, and the information contained in each feature is often “cross-informative” [59], which affects the final judgement. In this paper, SPA was used to eliminate the features with overlapping information and select numerous optimal features with less mutual redundancy for modelling to simplify the data and reduce the complexity of the model. The accuracy evaluation results of the model show that the sensitive feature variables selected by SPA can meet the needs of pest damage level identification.

**Figure 7:**
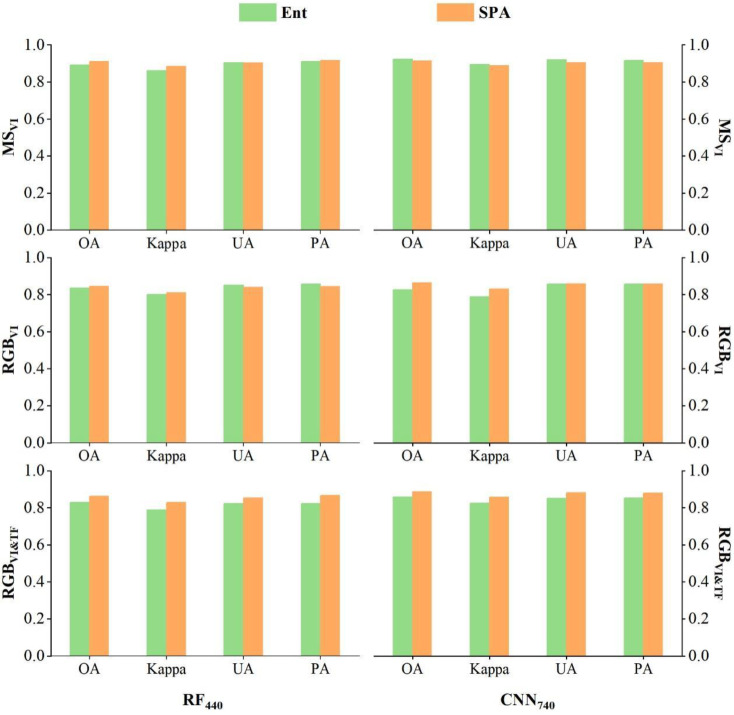
Modelling accuracy of entire features and sensitive features to different levels of trees.

To reveal the effect of SPA in feature screening more intuitively, this paper used all MS_VI_, RGB_VI_, and RGB_VI&TF_ features based on 440 and 740 samples to construct models (Ent-RF_440_ and Ent-CNN_740_, respectively) to compare them with the recognition effect of SPA’s sensitive features, and the results are shown in Figure 7. In both the RF and CNN categories, the SPA-based models not only did not decrease but even improved in accuracy compared with the models based on all the features, and, especially in the models based on the RGB_VI&TF_ features, all the accuracy metrics improved. For example, in model RF_440_, when RGB_VI&TF_-SPA was compared with RGB_VI&TF_-Ent, the OA, Kappa, UA, and PA were improved by 0.0.63, 0.0416, 0.032, and 0.0446, respectively; in model CNN_740_, with RGB_VI&TF_-SPA being compared with RGB_VI&TF_-Ent, the OA, Kappa, UA, and PA improved by 0.027, 0.0311, 0.0306, and 0.0272, respectively. The reason for this may have been that the information contained in the RGB_VI&TF_-Ent feature set had a high degree of redundancy, which affected the accuracy of the model.

Thus, the performance of various models proves that SPA can reduce the dimensionality of the data and reduce the amount of cumbersome information in the features, a step which, in our research, could effectively extract the features which were sensitive and meaningful to the degree of pest damage and improve the model’s stability and accuracy.

### 4.2. Differences in Recognition Accuracy for Different Damage Levels

The confusion matrix of RGB_VI&TF_-RF_440_ and RGB_VI&TF_-CNN_740_ revealed (Figure 6) that the model had a higher accuracy in recognizing health and severity levels and a slightly lower recognition accuracy for mild and moderate levels, a matter which is especially significant from the perspective of UA. This is because the difference in canopy colour caused by abnormal chlorophyll and water contents in needles is more obvious in healthy and severely damaged larch than in mildly and moderately damaged larch, which are green and grey–black, respectively, and in these cases, the features of red, green, and blue channels captured by RGB sensors are more prominent and easier to recognise, while the canopy colours of larch with mild and moderate levels of damage are more similar to one another (between yellow and red), meaning that the difference in the features reflected by the RGB sensor is small, and the RGB feature values of some sample trees with mild- and moderate-level damage appear similar, resulting in a lower prediction accuracy. In addition, *Erannis jacobsoni* Djak usually lays its eggs beneath the humus layer, and it feeds in a manner starting from the lower part of the larch and climbing upwards. This has a certain probability of causing a difference in the appearance of the upper (green) and lower (yellow) colours of larch at the same time. This leads to a situation in which a field survey classified a tree as having mild damage by the average rate of leaf loss, but the UAV orthophoto can only capture the green and healthy canopy of the upper part of the forest and cannot penetrate deeper to obtain information on the lower canopy, resulting in an incorrect classification. When the pest gradually eats from the lower part of the tree to its upper part, the appearance of the upper canopy of some trees starts to change, and the lower dead needles have a certain probability of regrowing (appearing green), resulting in a moderate degree of field division, and the colour information reflected to the UAV sensor in this case is a grey colour, characterizing a heavy degree of damage, thus causing recognition errors. This is consistent with the findings (UA, Health: 0.86, Mild: 0.02, Severe: 0.6, Dead:0.98) of Megat et al. when monitoring the extent of damage to eucalyptus trees from pests and diseases [60].

In the field survey, some needles of affected trees had a green or yellow semigloss form. Such needles were defined as damaged needles in the calculation of leaf loss rate and, thus, were classified as severely damaged from the perspective of the leaf loss rate but were identified as moderate or mild by the RGB colour features of the UAV camera, which explains why the PA of RGB_VI&TF_-CNN_740_ identified the severity damage as low, at 0.7872.

### 4.3. The Damage Level Recognition Potential of RGB_VI&TF_

The recognition of *Erannis jacobsoni* Djak pests by multispectral and RGB features revealed that MS_VI_-based models consistently outperformed RGB_VI_ models, similar to the results of most pest and disease studies [61]. To this end, the pest was recognised by combining RGB_VI_ and RGB image-derived texture features RGB_TF_ to create a new feature set, called RGB_VI&TF_. Finally, the recognition of RGB_VI&TF_ was improved compared with RGB_VI_, and the model accuracy was closer to that of MS_VI_, which proved the importance of TF in pest monitoring. This is because the study with RGB_VI_ only was based on the assumption that pixels are independent, meaning that any spatial relationship between neighbouring pixels was not considered in the preprocessing process [62], which may have affected the results due to “pretzel noise” in the classification image. TF is a common visual phenomenon—a local structure or arrangement rule which recurs in an image—which can reflect the intrinsic characteristics of the surface of the feature, does not vary with RGB reflection brightness, can characterise the spatial patterns and details between forest pixels [54], suppresses the “same spectrum, different object” and “same object, different spectrum” phenomena between forest canopy and understory vegetation spectra, and has a better robustness to factors such as illumination and shadows, thus providing information in our study which could not be explored by RGB_VI_. RGB_VI&TF_ contained not only RGB reflection information but also image texture information, capturing features related to the level of pest damage from different directions and containing more comprehensive disaster information so that model performance was significantly improved. This is consistent with the research results of Liu et al., combining the RGB vegetation index and texture features to estimate plant biomass to evaluate the health status of plants [63]. The combined mode of RGB_VI_ and RGB_TF_ in this experiment showed excellent capability and provided a new method for pest control research with low costs and a high efficiency.

In addition, the contribution ranking in Figure 5 shows that the main information-providing variable is RGB_VI_, not RGB_TF_, so we hypothesised that RGB_TF_ can provide supplementary information in pest monitoring studies but cannot be applied alone. To test the above hypothesis, only RGB_TF_ was used to complete the discrimination of pest damage levels. The results showed that the discrimination accuracy of RGB_TF_-RF_440_ and RGB_TF_-CNN_740_ was lower than that of the other models, and the optimal OA and Kappa did not reach 0.55, which made it difficult for the model to meet the experimental demands. This suggests that RGB_TF_ can only be involved in the pest recognition model as an auxiliary variable in combination with RGB_VI_. This is in agreement with the results of Zhou et al. in a vegetation recognition study [64].

### 4.4. Model Application

In this paper, traditional machine learning RF and deep learning CNN were utilised as algorithms for model construction. When using all the samples for modelling, CNN showed better results compared to RF, a finding which is consistent with our previous research and Kumar et al.’s findings on using deep learning for diagnosing plant early blight and late blight [38,65]. In addition, we found that the size of the sample had an impact on the model construction process, so we tested its performance, and an optimal model was obtained by changing the sample size. The RF model showed an inverted U-shaped trend in accuracy as the sample size increased, and the highest accuracy occurred when the sample size was 440, which was then the sample size set in the optimal model. The accuracy of the CNN model continued to increase as the sample size increased, and the optimal model was obtained when the sample size reached 740. The reason for this may have been that there was, inevitably, feature noise in the data due to the influence of shadows or backgrounds and label noise due to inconsistencies between the upper and lower canopies of the affected trees, leading to a gradual increase in the number of outliers in the process of increasing the sample size, which had a certain impact on the RF model and caused a decrease in accuracy and performance [66]. While CNN in deep learning uses local correlations in the process of convolution, it has some robustness in the face of outliers [67], and the model’s stability does not fluctuate significantly; thus, its accuracy improves as the sample size increases.

Of course, the CNN model has a sample size requirement, meaning that more samples are needed to train a more stable model [68]; therefore, deep learning algorithms outperform traditional machine learning algorithms when the sample size is sufficient [69,70]. CNN can be used as a first choice to identify the level of damage of *Erannis jacobsoni* Djak pests. However, field sampling is difficult, and a sufficient number of samples may not be used in every trial, so the RF algorithm can be used instead in the absence of samples and can effectively recognise the presence of pests.

### 4.5. Limitations and Prospects

The experiment showed that the accuracy of the MS_VI_-based model could meet the requirements for the identification of the damage level of *Erannis jacobsoni* Djak [71], which was expected. However, its high acquisition cost limits the progress of some important studies. In contrast, RGB images are cheaper to acquire than multispectral images, and our research’s results on the combined RGB_VI&TF_ indices obtained by RGB images are close to those of MS_VI_, making low-cost and high-precision pest identification possible.

In this paper, through the experience of previous studies, field survey parameters and UAV images of the test area at the end of June were used as the basic data sources for *Erannis jacobsoni* Djak pest monitoring. However, the timing of canopy colour change after each insect infestation varied, a phenomenon which was related to insect population density, tree genetics, host vigour, and environmental conditions [72,73]. In turn, long-term field observations were needed to collect drone images and ground data at appropriate times for the accurate identification of pests [48].

Since the focus of this paper was to investigate the potential of RGB images in pest identification, only 22 RGB vegetation indices and RF and CNN algorithms [37], which are widely used by scholars, were selected to obtain more satisfactory results in the construction of the model. If we were to refer to some more meaningful RGB vegetation indices and frontier algorithms on this basis, the accuracy and generalisation ability of the model would very likely improve, a matter which will be explored in our next experiments.

## 5. Conclusions

In this study, we used UAV images of an *Erannis jacobsoni* Djak outbreak area as the data source, used a combined model of RGB_VI_ and RGB_TF_ to construct a pest damage recognition model, and compared and analysed the experimental results with other models using MS_VI_ and RGB_VI_ to explore the potential of RGB_VI&TF_. This study confirmed that features derived from low-cost RGB images can essentially replace the multispectral identification of *Erannis jacobsoni* Djak pest damage levels. The experimental results were more optimistic: the accuracy of the RGB_VI_-based pest damage recognition model was very low compared with that of MS_VI_, but the results changed after combining it with RGB_TF_, and the accuracies of RGB_VI&TF_-RF and RGB_VI&TF_-CNN were significantly improved compared with those of RGB_VI_-RF and RGB_VI_-CNN and were close to those of MS_VI_-RF and MS_VI_-CNN. On this basis, the confusion matrix of the RGB_VI&TF_ model revealed that the model was extremely good at recognizing healthy and severely infested regions, while the detection accuracy of mildly and moderately infested regions was low, which was caused by pest habits and the orthorectification principle of UAV. In addition, SPA eliminated redundant and overlapping information in the data to provide effective input variables for the model, and the accuracy and suitability of the model constructed by SPA were improved compared with those of the models using all the features.

Using RGB_VI&TF_ can achieve the identification of the level of pest damage at a small scale, and the results of this model can meet the needs of the relevant forestry departments. This study also provides a reference example and a theoretical basis for subsequent low-cost, large-area pest monitoring and control with a high spatial and temporal resolution and forest ecosystem protection.

## Figures and Tables

**Table 1:** Damage level classification criteria.

Mark	1	2	3	4
Damage level	Healthy	Mild	Moderate	Severe
Leaf loss rate	0–5%	6–30%	31–70%	71–100%

**Table 2:** Input sensitive features of optimal RF models.

Feature Sets	Sensitive Features	Formula
MS_VI_-SPA_440_	2NLI	(NIR^2^ − g)/(NIR^2^ + g)
	GDVI	NIR − g
	GMNLI	1.5(NIR^0.5^ − g)/(NIR^0.5^ + g + 0.5)
	NDVIreg	(NIR − RE)/(NIR + RE)
	SI1reg	(g*RE)^0.5^
	SI1reg*	(r*RE)^0.5^
	TCARI	3[(RE − r) − 0.2(RE − g)(RE/r)]
RGB_VI_-SPA_440_	ExG	2 g − r − b
RGB_VI_-SPA_440_/RGB_VI&TF_-SPA_440_	B	B
	ExR	1.4R − G
	GBRI	G/B
	GCC	G/(R + G + B)
	R	R
	RBRI	R/B
	RGRI	R/G
	VDVI	(G − B − R)/(G + B + R)
RGB_VI&TF_-SPA_440_	GLA	(2G − R − B)/(2G + R + B)
	CIVE	0.441r − 0.881 g + 0.3856b + 18.78745
	Mean	$\sum_{i,j=0}^{N-1}i\times P_{i,j}$
	Dis	$\sum_{i,j=0}^{N-1}i\times P_{i,j}\left|i-j\right|$
	Ent	$\sum_{i,j=0}^{N-1}i\times P_{i,j}(-lnP_{i,j})$
	SM	$\sum_{i,j=0}^{N-1}i\times P_{i,j}^{2}$

In the MS_VI_, b, g, r, RE, and NIR represent the spectral reflectance at the blue, green, red, red-edge, and near-infrared bands, respectively. In the RGB_VI_ and RGB_TF_, R, G, and B represent the reflectance at the red, green, and blue channels, respectively. “*i*, *j*” represents row number (*i*) and column number (*j*) in the matrix *P*; “*N*” represents row number or column number in *P*; and “*P_i_*_,*j*_” represents cell I, a normalised value in *J*.

**Table 3:** Input sensitive features of optimal CNN models.

Feature Sets	Sensitive Features	Formula
MS_VI_-SPA_740_	2NLI	(NIR^2^ − g)/(NIR^2^ + g)
	GMNLI	1.5(NIR^0.5^ − g)/(NIR^0.5^ + g + 0.5)
	MTVI2	1.5[1.2(NIR − g) − 2.5(r − g)]/[(2NIR + 1)^2^ − (6NIR − 5r^0.5^) − 0.5]^0.5^
	Int2reg*	(g + r + RE)/2
	NDSIreg	(RE − NIR)/(RE + NIR)
	RECI	(NIR/RE) − 1
	SCCI	100(lnNIR − lnr)/[(NIR − r)/(NIR + r)]
	SI1reg	(g*RE)^0.5^
	SI1reg*	(r*RE)^0.5^
	SI2reg	(g^2^ + RE^2^ + NIR^2^)^0.5^
RGB_VI_-SPA_740_	GLA	2 g − r − b
RGB_VI_-SPA_740_/RGB_VI&TF_-SPA_740_	GB	g − b
	GBRI	G/B
	RBRI	R/B
RGB_VI&TF_-SPA_740_	RGRI	0.441r − 0.881 g + 0.3856b + 18.78745
	Mean	$\sum_{i,j=0}^{N-1}i\times P_{i,j}$
	Dis	$\sum_{i,j=0}^{N-1}i\times P_{i,j}\left|i-j\right|$

In the MS_VI_, b, g, r, RE, and NIR represent the spectral reflectance at the blue, green, red, red-edge, and near-infrared bands, respectively. In the RGB_VI_ and RGB_TF_, R, G, and B represent the reflectance at the red, green, and blue channels, respectively. “*i*, *j*” represents row number (*i*) and column number (*j*) in the matrix *P*; “*N*” represents row number or column number in *P*; and “*P_i_*_,*j*_” represents cell *I*, a normalised value in *J*.

**Table 4:** Accuracy evaluation of RF model for trees with different damage levels.

	OA			Kappa		
			Features Set			
Size of Simple Trees	MS_VI_-SPA	RGB_VI_-SPA	RGB_VI&TF_-SPA	MS_VI_-SPA	RGB_VI_-SPA	RGB_VI&TF_-SPA
140	0.7429	0.6286	0.7714	0.6927	0.5663	0.7276
240	0.7000	0.6333	0.7333	0.6487	0.5788	0.6836
340	0.8706	0.8353	0.8353	0.8372	0.7964	0.7964
440	**0.9091**	**0.8455**	**0.8636**	**0.8843**	**0.8092**	**0.8295**
540	0.8889	0.837	0.8593	0.8589	0.7987	0.8241
640	0.8812	0.825	0.825	0.8515	0.7864	0.7864
740	0.8432	0.8324	0.8270	0.8050	0.7935	0.7887
840	0.8	0.8286	0.8143	0.7568	0.7911	0.7708

Bold digits indicate the highest model accuracy in each features set.

**Table 5:** Accuracy evaluation of CNN model for trees with different damage levels.

	OA			KAPPA		
			Features Set			
Size of Simple Trees	MS_VI_-SPA	RGB_VI_-SPA	RGB_VI&TF_-SPA	MS_VI_-SPA	RGB_VI_-SPA	RGB_VI&TF_-SPA
140	0.7714	0.6286	0.7714	0.7255	0.5604	0.7282
240	0.8	0.6833	0.7833	0.7581	0.6329	0.7376
340	0.8235	0.7412	0.8353	0.7825	0.6923	0.7969
440	0.8273	0.7636	0.8455	0.7860	0.7149	0.8080
540	0.8519	0.8148	0.8519	0.8157	0.7703	0.8153
640	0.8875	0.825	0.8688	0.8591	0.7859	0.8370
740	**0.9135**	**0.8649**	**0.8865**	**0.8892**	**0.8306**	**0.8565**
840	0.8857	0.8381	0.8286	0.856	0.7996	0.7889

Bold digits indicate the highest model accuracy in each features set.

## Data Availability

All data are provided in the manuscript.
